# The Role of Reactive Species on Innate Immunity

**DOI:** 10.3390/vaccines10101735

**Published:** 2022-10-17

**Authors:** Celia María Curieses Andrés, José Manuel Pérez de la Lastra, Celia Andrés Juan, Francisco J. Plou, Eduardo Pérez-Lebeña

**Affiliations:** 1 Hospital Clínico Universitario of Valladolid, Avenida de Ramón y Cajal 3, 47003 Valladolid, Spain; 2 Institute of Natural Products and Agrobiology, CSIC-Spanish Research Council, Avda. Astrofísico Fco. Sánchez 3, 38206 La Laguna, Spain; 3 Cinquima Institute and Department of Organic Chemistry, Faculty of Sciences, Valladolid University, Paseo de Belén 7, 47011 Valladolid, Spain; 4 Institute of Catalysis and Petrochemistry, CSIC-Spanish Research Council, 28049 Madrid, Spain; 5 Sistemas de Biotecnología y Recursos Naturales, 47625 Valladolid, Spain

**Keywords:** reactive species, ROS, RNS and RHS, innate immunity, antimicrobial

## Abstract

This review examines the role of reactive species RS (of oxygen ROS, nitrogen RNS and halogen RHS) on innate immunity. The importance of these species in innate immunity was first recognized in phagocytes that underwent a “respiratory burst” after activation. The anion superoxide ^•^O_2_^−^ and hydrogen peroxide H_2_O_2_ are detrimental to the microbial population. NADPH oxidase NOx, as an ^•^O_2_^−^ producer is essential for microbial destruction, and patients lacking this functional oxidase are more susceptible to microbial infections. Reactive nitrogen species RNS (the most important are nitric oxide radical -^•^NO, peroxynitrite ONOO^—^ and its derivatives), are also harmful to microorganisms, including bacteria, viruses, and parasites. Hypochlorous acid HOCl and hypothiocyanous acid HOSCN synthesized through the enzyme myeloperoxidase MPO, which catalyzes the reaction between H_2_O_2_ and Cl^−^ or SCN^−^, are important inorganic bactericidal molecules, effective against a wide range of microbes. This review also discusses the role of antimicrobial peptides AMPs and their induction of ROS. In summary, reactive species RS are the heart of the innate immune system, and they are necessary for microbial lysis in infections that can affect mammals throughout their lives.

## 1. Introduction

The immune system can be divided into innate and acquired immunity, which are closely related [1]. Innate immunity is possessed by all types of multicellular organisms and is a primitive prophylactic system in which macrophages, neutrophils, and dendritic cells are primarily responsible for its functioning [2]. In addition, the following are also involved: antimicrobial peptides, natural antibodies, the complement system, NK cells, and gamma delta T lymphocytes [3].

Cells involved in innate immunity recognize foreign substances such as bacteria with toll-like receptors (TLR) and regulate the activation of other cells by the production of various cytokines [4]. There are cells, such as phagocytes, that can invade bodies in a process in which the cell uses its plasma membrane to engulf the large particle, giving rise to an internal compartment called a phagosome, and then activating the acquired immunity system by presenting a portion of the phagocytosed and digested foreign substances from its membrane surface. In the recognition and response process of phagocytosis, reactive oxygen species ROS and reactive nitrogen species RNS are produced [5].

Physiological levels of reactive species (ROS and RNS) are important in cellular signaling, but higher concentrations and prolonged exposure can fight infections by damaging important microbial biomolecules [6]. The chemical changes mediated by reactive species RS are detrimental to cell function because they cause oxidation and nitration, altering the structures of cellular proteins, DNA, and lipids, and impairing their normal function. ^•^NO_2_ and ^•^OH can modify proteins by reacting with tyrosine, tryptophan, cysteine, and methionine residues, promoting hydroxylation and nitration in peptides and proteins, impairing their normal function [7].

In the event of bacterial and fungal infection, rapid generation of ROS is essential for host defense. Therefore, ROS generation is important for effective antimicrobial defense, which can prevent inflammation and excessive tissue injury. The human body generates approximately 5 g ROS/day mainly from the leakage of the electron transport chain during oxidative phosphorylation, in the inner membrane of the mitochondrial matrix [8]. **^•^**O_2_^−^ and hydrogen peroxide H_2_O_2_ are the two primary products of this leakage. However, the generation of any O_2_ derivative species is dynamically balanced. These radical species have a dual role, at the physiological level they are cellular signalers, and when there is an imbalance between their production and the antioxidant system they are involved in several harmful biological processes, such as protein denaturation and lipid peroxidation.

ROS are generated on the membranes of the endosome of the phagocytosing cells, with the involvement of NOx [9]. Superoxide anion ^•^O_2_^−^ is also produced in the mitochondrial matrix at complexes I to IV during the mitochondrial respiration process [10]. In addition to mitochondria, ROS are produced by a variety of enzymes such as NOx, xanthine oxidase, nitric oxide synthase NOS, and in other cell organelles such as the endoplasmic reticulum, peroxisomes, and cytosol [11]. ^•^O_2_^−^ is unstable and cannot pass through membranes but is rapidly converted to hydrogen peroxide H_2_O_2_, a membrane-permeable specie [12]. H_2_O_2_, in biochemical reactions, produces the hydroxyl radical ^•^OH + ^−^OH, highly reactive in the mitochondrial matrix [13].

ROS can also trigger the pathogen defense of phagocytes by non-oxidative means, such as autophagy, receptor signaling, extracellular trapping, and originating lymphocyte action. For example, H_2_O_2_ can also modulate genes expression by epigenetic modification and activate transcription factors such as AP-1, NRF2, CREB, HSF1, HIF-1, TP53, NF-κβ, NOTCH, SP1, SCREB-1 and FOXO family [14,15,16,17,18,19,20,21,22].

Nitric oxide ^•^NO is a ubiquitous cellular signaling molecule, found in a variety of cell types, including vascular endothelium, platelets, macrophages, and neuronal cells [23]. In the cardiovascular system, ^•^NO determines the basal vascular tone and myocardial contractility, inhibits platelet aggregation, limits endothelial adhesion of leukocytes, and regulates myocardial contractility, playing a role in the etiology of cardiovascular disorders: atherosclerosis, hypertension, reperfusion injury and myocardial depression associated with sepsis and septic shock [24]. Vascular endothelial cells continuously produce ^•^NO and this basal release regulates the vascular tone. The oxidation of the terminal guanidino-nitrogen atoms of L-arginine produces ^•^NO [25].

Peroxynitrite ONOO^—^ is a potent oxidizing and nitrating agent, with a short half-life of about 10^−2^ s [26]. Its derivatives induce lipid peroxidation, inactivation of enzymes and proteins, and mitochondrial dysfunction, among others. ONOO^—^ plays an important role in the destruction of foreign pathogens by cells such as macrophages [27]. If its production is deregulated, it contributes to cardiovascular, neurological diseases, and cancer [7]. Secondary reactions of peroxynitrite decomposition produce ^•^NO_2_, ^•^OH, and ^•^CO_3_^—^.

The enzyme myeloperoxidase MPO is a hydrogen peroxide oxidoreductase, present in macrophages, in different biological fluids (saliva, synovial fluid, and semen, among others), and in different tissues (heart, kidney, skin, liver, and placenta) [28]. The most common sources are neutrophils, where the enzyme is located at the lysosomal level [29]. The reaction catalyzed by MPO is the oxidation of the Cl^−^ anion by H_2_O_2_ to give hypochlorous acid HOCl, a very reactive and oxidizing agent, which can also act as a chlorinating agent and is the main strong oxidant generated by neutrophils in appreciable quantities [30].

**Figure 1:**
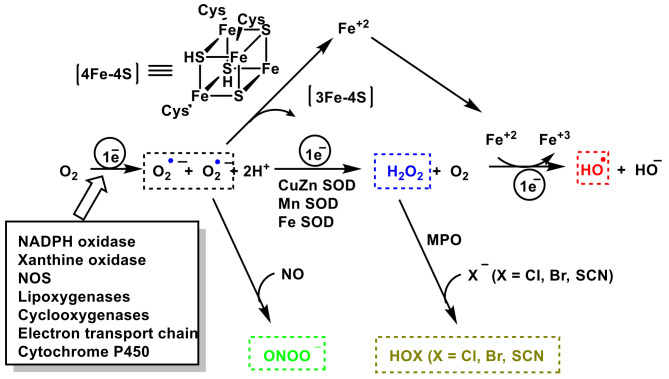
Chemical and enzymatic reactions generating ROS, RNS, and RHS.

Another known oxidizing agent involved in the innate immune system is the hypothiocyanite anion OSCN^−^ and the hypothiocyanic acid conjugate base HOSCN (a weak acid with a dissociation constant of pKa = 5.3). It is an organic compound that contains the functional group SCN^−^. OSCN^−^ is formed by peroxidase enzyme catalysis of hydrogen peroxide and thiocyanate: H_2_O_2_ + SCN^−^ → OSCN^−^ + H_2_O. Hypothiocyanite occurs naturally as an antimicrobial agent in the human respiratory tract. OSCN^−^ is harmless to cells in the human body but is cytotoxic to bacteria, so it has been widely investigated for its capabilities as an alternative antibiotic agent. The chemical and enzymatic scheme for the generation of ROS, RNS, and RHS is represented in Figure 1.

## 2. Function and Features of Immunity and Innate Immune System

**Figure 2:**
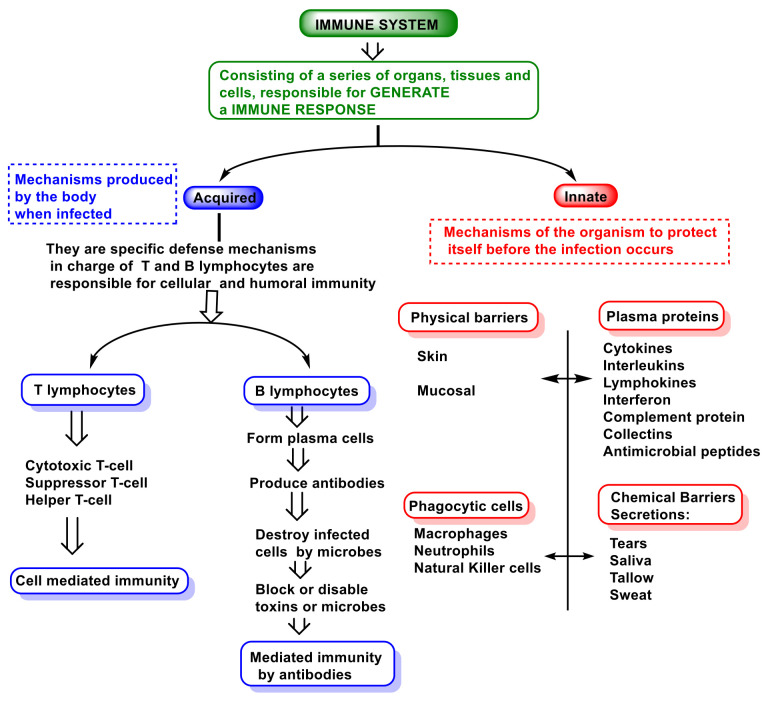
Innate and adaptive immune responses.

The immune system involves cells, organs, proteins, and tissues throughout the body, and it comprises components such as leukocytes, spleen, bone marrow, lymphatic system, thymus, tonsils, adenoids, and appendix [31]. There are three types of immunity in humans: innate, adaptive, and passive. Figure 2 is a representation of the immune system.

Innate immunity is the immunity that people are born with and provides a certain level of immunity that attacks invaders from day one. This innate immunity is the first line of defense against pathogens and includes the skin and mucous membranes of the throat and gut. Innate is non-specific immunity and is an ancient evolutionary defense strategy found in plants, fungi, animals, and primitive multicellular organisms [32].

Adaptive immunity involves specific immune cells and antibodies, and it can prevent disease in the future by remembering what those substances look like and mounting a new immune response, and is carried out by lymphocytes.

Vertebrates, exclusively, have adaptive immunity, which can recognize and destroy specific substances [33]. The adaptive immune response provides vertebrates with the ability to recognize and remember specific pathogens, generating immunity and delivering increasingly potent responses to the re-encountered pathogen. Adaptative immunity includes two parts: one is called humoral and involves a variety of substances found in the body’s humors or fluids, which interfere with the growth of pathogens or clump them together so that they can be eliminated from the body.

The antibody and cell-mediated immune responses are carried out by different classes of lymphocytes, called B cells and T cells, respectively. B cells are activated to secrete antibodies, a type of protein called immunoglobulins, which circulate through the bloodstream and penetrate other body fluids, eventually binding specifically to the foreign antigen that stimulated their production. Antibody binding inactivates viruses and microbial toxins by blocking their ability to bind to receptors on host cells. Antibody binding also marks invading pathogens for destruction by making it easier for the phagocytes of the innate immune system to ingest them [34]. The cell-mediated response is carried out by phagocytes, which ingest and degrade pathogens, as well as by natural killer cells that destroy certain cancer cells [35].

In contrast, adaptive immunity is also responsible for allergic reactions and the rejection of transplanted tissues, which it recognizes as a foreign invader [36].

Passive immunity is a type of temporary immunity that is derived from another person. For example, a newborn receives antibodies from the mother through the placenta before birth and in breast milk after birth. This passive immunity protects the baby from some infections during the first years of life.

Innate and adaptive systems work together to provide vertebrates with increased resistance to micro-organisms, parasites, and potential intruders that may cause harm.

In innate immunity, invaders are identified by pattern recognition receptors that distinguish molecules expressed on microbial surfaces, called pathogen-associated molecular patterns (PAMPs). A second trigger is molecules released from broken or damaged cells, called damage-associated molecular patterns (DAMPs) [37].

Polymorphonuclear leukocytes PMNs recognize secreted molecules produced by bacteria, including peptidoglycan, lipoproteins, lipoteichoic acid, lipopolysaccharide (LPS), CpG-containing DNA, and flagellin. Peptidoglycan recognition protein (PGRP) plays a role in the neutrophil killing of Gram-positive bacteria [38], inhibiting their growth.

A class of pathogen recognition receptors is toll-like receptors (TLRS), a family of at least 10 different receptors found on the surface or in the cytoplasm of cells such as macrophages, intestinal epithelial cells, and mast cells, and which are located on the surface or the membrane of endosomes [39]. Toll receptors bind to PAMPs on extracellular bacteria, such as lipopolysaccharides, flagellin, and lipoproteins [40]. Cytoplasmic TLRs bind to the nucleic acids of intracellular viruses [41]. Once bound to these ligands, TLRs trigger the production of inflammatory cytokines such as interleukin IL-β1 or the tumor necrosis factor TNF-α, triggering what is termed acute inflammation [42].

The presence of inflammatory chemokines controls the recruitment of effector leukocytes in infections, inflammation, tissue injury, and tumors, and has a broad cellular selectivity, acting on cells of both the innate and adaptive immune systems [43]. In this process participates integrins and transmembrane cell adhesion molecules, which regulate cellular growth, proliferation, migration, cellular signaling, cytokine activation, and its release. Therefore, they play important roles in cell proliferation and migration, apoptosis, and tissue repair, as well as in all processes critical to inflammation, infection, and angiogenesis [43,44].

Acute inflammation is the central feature of innate immunity and it is the subsequent step in the early detection of invading organisms or damaged tissues [45]. The inflammatory response is characterized by several features: reddening of the skin (due to increased blood circulation), warmth or increased temperature (sensation of heat around a local infection or systemic fever), swelling of affected tissues (in the throat during the common cold or in joints affected by rheumatoid arthritis), mucus production (runny nose or cough), pain (in sore joints or in the throat) and even possible dysfunction of affected organs and tissues [1]. Inflammation guarantees that leukocytes converge in large numbers towards the site of microbial invasion, attracting these cells from the bloodstream and inducing them to migrate through the tissues to the invasion site [46].

The key to an effective innate response is the rapid recognition of the invasion, for which there are several types of sentinel cells. The most important are macrophages, dendritic cells, and mast and innate lymphoid cells [47]. The first three possess pattern recognition receptors and can detect the presence of PAMPs and DAMPs, so they send a signal through the nuclear factor NF-κβ, to produce cytokines such as IL-1, interferon IFN-α and TNF-α [48]. Molecules such as histamine, leukotrienes, prostaglandin, and specialized peptides are released to initiate the inflammatory process. Three main populations of leukocytes can eliminate invaders: (i) neutrophils, which are particularly effective at killing invading bacteria by engulfing them, activating the respiratory burst, and generating lethal oxidative molecules such as hydrogen peroxide and hypochlorite ions, which kill most invading bacteria; (ii) eosinophils, specialized killers of invading parasites, which for example contain enzymes optimized to kill helminth larvae; and (iii) M1-like macrophages, capable of migrating to areas of microbial invasion more slowly than granulocytes, but able to maintain sustained and effective phagocytosis [49]. They contain the lethal antimicrobial ^•^NO and can kill neutrophil-resistant organisms. If inflammation activates macrophages, they secrete a cytokine called IL-23, which acts on the Th17 cell subset, secreting IL-17, which attracts neutrophil granulocytes to sites of inflammation, infection, and tissue damage [50].

Mammals possess at least four populations of innate lymphoid cells ILCs that participate in innate immunity: natural killer (NK) cells, ILC1s, ILC2s, ILC3s, and lymphoid tissue inducer cells [51]. NK natural killer cells are innate lymphoid cells optimized to kill virus-infected cells, and can even kill abnormal cells, which do not express MHC class I major histocompatibility complex molecules [52]. Group I of innate lymphoid cells are found in large numbers in the intestinal wall and secrete macrophage-activating cytokines, playing a key role in antiviral immunity [53]. Group II of innate lymphoid cells are distributed throughout the body and secrete cytokines important for anti-parasitic immunity [54]. Group III of innate lymphoid cells act as Th17 cells and promote inflammation by releasing IL-17 [55]. Lymphoid tissue-inducing cells are hematopoietic and have critical roles in the immune system, both in the embryonic and adult stages [56]. These cells fulfill the following four functions: defense against pathogens, surveillance of tumorigenesis, regulation of homeostasis, and tissue remodeling [57].

When neutrophils arrive at the site of invasion, they bind to invading bacteria and ingest them by phagocytosis, a process mediated by a metabolic pathway called a respiratory burst, which generates oxidative species such as H_2_O_2_ and hypochlorous acid HOCl [58]. In contrast, the energy reserves of neutrophils are minimal, and they can only perform a few phagocytic events before they decay. Once the invading microbes are successfully eliminated, the body must repair the damage and eliminate cellular debris and dying cells through the work of macrophages, which originate from monocytes in the blood [59]. Macrophages are attracted to sites of microbial invasion and tissue damage by chemokines, DAMPs, and PAMPs, help kill invaders, remove toxic waste produced in tissues and destroy remaining neutrophils. M1-like macrophages complete the destructive process and are optimized for microbial destruction, while M2-like macrophages are optimized for the removal and repair of damaged tissues [60].

Besides the role played by neutrophils, there is also a parallel mechanism called NETosis, related to the formation of neutrophil extracellular traps (NETs). Various pathogens, antibodies and immune complexes, cytokines, and other physiological stimuli can trigger NETosis. Its induction depends on ROS, the main source being NOx [61]. NOx activation depends on increased Ca^2+^ concentration in the cytoplasm and, in some cases, on the generation of mitochondrial ROS. NETosis results in the release of granule components into the cytosol, histone modification leading to chromatin decondensation, destruction of the nuclear envelope, as well as the formation of pores in the plasma membrane. Two forms of NETosis have now been described: classical or suicidal NETosis (leading to cell death), and vital NETosis, where the cell retains its viability and many of its effector functions [62].

Classical NETosis is a special form of programmed cell death (PCD), characterized by the release of granule components into the cytosol. Several features of apoptosis, necroptosis, pyroptosis, autophagy, and secondary necrosis are inherent to this form of NETosis. Mitochondrial ROS are involved in NOx activation and in the induction of classical NETosis by various stimuli [63,64].

Vital NETosis helps contain local infections by allowing PMNs to rapidly release NETs and continue to phagocytose live bacteria. In addition, live PMNs that release NETs manage to maintain their membrane integrity, thereby imprisoning the captured bacteria [65,66].

Alongside ROS production, macrophages also employ several directly antimicrobial mechanisms, such as the generation of RNS in the phagosome, and the delivery of cathepsins and other hydrolases into maturing phagosomes [67]. Other indirect antimicrobial mechanisms include: (i) activation of inflammasomes and (ii) secretion of cytokines and chemokines [68]. These mechanisms help orchestrate subsequent innate and adaptive immune responses, as well as major histocompatibility complex MHC-dependent presentation of pathogen-derived antigens [69].

In parallel with acute inflammation, the body has other innate defenses as tissues contain a variety of antimicrobial peptides. These include antimicrobial peptides, such as defensins or cathelicidins, enzymes such as lysozyme that kill many Gram-positive bacteria, and iron-binding proteins such as hepcidin or haptoglobin that prevent the growth of bacteria by depriving them of vital iron [70]. The most important of these defenses is the complement system, a group of about 30 proteins that work together to eliminate invading microbes by covalently and irreversibly binding two proteins called C3 and C4 to microbial surfaces. Once bound, they can lyse microbes via the C5–C8 complex formation and the polymerization of C9 protein forming a membrane attack complex MAC, or participates as opsonins, promoting a quickly and efficiently phagocytosis by leukocytes [71].

This system can be activated in three ways:

(i) The so-called alternative pathway is activated by the presence of bacterial surfaces that can bind complement protein C3. C3-coated bacteria are rapidly and efficiently phagocytosed and destroyed. C3 can activate other complement components by inducing a protein called C9 to insert itself into the cell walls of bacteria, causing them to rupture;
(ii) A second pathway of complement activation is triggered when bacterial surface carbohydrates bind to a mannose-binding lectin (MBL), collectin 11 (CL-K1), and ficolins (Ficolin-1, Ficolin-2, and Ficolin-3). Its activation leads to C4 and C2 activation by their serine-proteases; or
(iii) The classical complement pathway is initiated by antigen-antibody complexes with the antibody isotypes IgG and IgM. Upon activation, several proteins are recruited to generate C3 convertase, which cleaves the C3 protein. The C3b component of cleaved C3 binds to the C3 convertase to generate the C5 convertase, which cleaves the C5 protein. The cleaved products attract phagocytes to the site of infection and mark target cells for elimination by phagocytosis. C5 convertase initiates the terminal phase of the complement system, resulting in the assembly of the MAC membrane attack complex, creating a pore in the target cell membrane, and inducing its lysis [72].

Because of its potential to cause severe tissue damage, the activation of the complement system is carefully controlled through multiple complex regulatory pathways [73].

The complement system plays an important role in mediating tissue injury following the triggering of oxidative stress. Collard et al., 2000, investigated the role of mannose-binding lectin (MBL) and the lectin complement pathway (LCP) in mediating complement activation following endothelial oxidative stress and observed that the LCP lectin complement pathway mediates complement activation following tissue oxidative stress. Thus, they suggest that inhibition of MBL may represent a novel therapeutic strategy for ischaemia/reperfusion injury and other complement-mediated disease states [74].

## 3. Role of Superoxide Anion ^•^O_2_^−^ and Hydrogen Peroxide H_2_O_2_ on Innate Immunity

**Figure 3:**
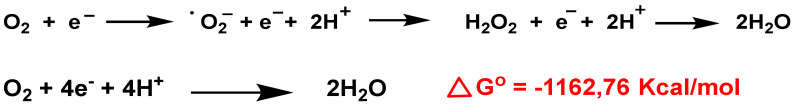
O_2_ reduction chain to ^•^O_2_^−^, H_2_O_2_ and H_2_O.

O_2_^−^ anion is a by-product of mitochondrial respiration and a crucial element of the innate immune defense system. Biochemically, **^•^**O_2_^−^ is generated from two main sources: in the respiratory chain in the mitochondrial matrix and via nicotinamide adenine dinucleotide phosphate. In the electron transport chain, protons introduced by ATP synthase reduce molecular O_2_ to ^•^O_2_^−^ anion, H_2_O_2,_ and H_2_O [75], Figure 3. Consecutive reduction of O_2_ with H^+^ and e^—^ have a negative Gibbs energy, so it occurs spontaneously, with a ∆Go ≤ 0. The Gibbs free energy is used to calculate the maximum amount of work that can be done by a thermodynamically closed system, with temperature and pressure being constant, and is a necessary condition in processes such as chemical reactions.

In the cytosolic SOD-Cu/Zn (it contains Cu and Zn in the catalytic site), SOD transforms the ^•^O_2_^−^ to O_2_ reducing Cu(II) to Cu(I). Another ^•^O_2_^−^ molecule causes the oxidation of Cu(I) to Cu(II), producing an H_2_O_2_ molecule. Zn is monovalent and it only stabilizes the enzyme. The catalytic cycle of Mn SOD is similar, with Mn in the oxidation-reduction reactions, transiting between Mn(III)) and (Mn(II).

Operative roles of H_2_O_2_ during inflammation have been observed, modulating protein function by reversible chemical modification of protein thiols [76]. H_2_O_2_ induces activation of factor nuclear NF-κβ (the factor that controls DNA transcription), including tyrosine phosphorylation of IkB and activation of IKK [77]. H_2_O_2_ can trigger the release of high mobility group 1 protein from macrophages, follow-on increase of proinflammatory stimuli [78].

Polymorphonuclear neutrophils PMN are a critical constituent of the innate immune system. In case of infection, neutrophils are rapidly recruited from the circulation and bone marrow stores by the host- and pathogen-derived components, priming these cells for enhanced antimicrobial activity [79]. One of the most potent biochemical attractants is the interleukin IL-8, produced by cells during the inflammatory process associated with infection [80]. Cells that produce interleukin IL-8 include monocytes, macrophages, mast cells, epithelial cells, keratinocytes, fibroblasts, endothelial cells, and even neutrophils themselves [81]. Bacteria also produce molecules that can directly attract neutrophils, e.g., N-formyl peptides [82]. Neutrophil “priming” is the ability to increase superoxide anion. In fact, this capacity is not limited to **^•^**O_2_^−^ production, but also to improved adhesion, phagocytosis, cytokine secretion, leukotriene synthesis, degranulation, and, ultimately, bactericidal activity. In this “priming” effect, neutrophils respond to the release of cytokines, chemokines, growth factors, and lipid-derived signaling molecules. In summary, neutrophils react increasing the release of **^•^**O_2_^−^ and inducing the expression of, among others, TNF-α, IFN-γ and -α, several interleukins, C2-ceramide, peroxynitrite, or diamide (thiol oxidizer) [83].

The combination of ROS from neutrophils and granule components is usually effective in killing most bacteria and fungi. PMNs are the most abundant leukocyte in humans and contain a battery of non-specific cytotoxic compounds, so their homeostasis is highly structured. Once neutrophil apoptosis occurs, these cells are eliminated by macrophages, and their apoptosis is accelerated following phagocytosis of bacteria, completing the termination of the infection and associated inflammation [84].

Ultimately, neutrophils use both O_2_-dependent and O_2_-independent mechanisms to kill micro-organisms [85]. Phagocytosis triggers the generation of **^•^**O_2_^−^ and other ROS and reactive species, such as hydrogen peroxide H_2_O_2_, hypochlorous acid HOCl, hydroxyl radical **^•^**OH and chloramines, as potent microbicidal agents [86]. In parallel, cytoplasmic granules fuse with phagosomes containing bacteria in a process known as degranulation, thereby enriching the vacuole lumen with antimicrobial peptides and proteases [87].

NOx catalyzes the reduction reaction of O_2_ to ^•^O_2_^−^ and/or H_2_O_2_ using NADPH as an electron donor and it is located extracellularly [88]. NOx is involved in pathogen clearance and the regulation of associated inflammation plays an important role in physiological and pathological conditions, such as acute lung injury and bacterial or fungal infections. NOx is electrogenic and allows electron transport across the plasma membrane (altering ionic currents) [89], induces apoptosis (mediating in physiological and pathological processes) [90], regulates cytokine production and T cell death [91], influences gene expression and promotes the formation of extracellular traps [92,93].

The significance of NOx and ROS production is exemplified by a rare inherited disorder known as chronic granulomatous disease CGD. Individuals with CGD have persistent bacterial and fungal infections due to defects in NOx [94].

Following NOx activation, there is a rapid expenditure of O_2_ in neutrophils, and this mechanism is called the “respiratory burst”. NOx activation by neutrophils occurs in response to stimuli such as formylated peptides, opsonized particles, integrin-dependent adhesion, and the binding of specific pathogen recognition receptors (e.g., dectin-1). SYK tyrosine kinase is a critical component of integrin signaling in neutrophils, mediating NOx activation [95]. SYK tyrosine kinase is a non-receptor kinase that was long considered to exclusively mediate receptor signaling in the adaptive immune response. However, recent studies indicate that it is also involved in innate immunity and non-immune functions. SYK mediates integrin signaling in neutrophils, macrophages, and platelets, signaling by P-selectin glycoprotein ligand 1 (PSGL1), as well as the development of osteoclasts [96]. SYK participates in the innate recognition of fungal and other microbial pathogens, as well as of tissue damage, by C-type lectins. SYK activation by C-type lectins activates the caspase-recruitment domain 9–B cell lymphoma and it is also required for NLR family, pyrin domain-containing 3 (NLRP3) inflammasome activation following fungal infection [97].

It is known that H_2_O_2_ forms naturally in living organisms and its attributed physiological role is the capability to induce bacterial killing. It has been estimated that, in lymphocytes, the half-life of the H_2_O_2_ is 1 ms while that of the anion **^•^**O_2_^−^ is 1 µs [98]. It is not a free radical, but it is a very important reactive form, generating the **^•^**OH radical in the presence of metals such as iron (Fenton reaction). The hydroxyl radical ^•^OH, the most powerful ROS oxidant, is formed during the Haber–Weiss reaction, by the Fenton reaction or by decomposition of peroxynitrite, and has a very short half-life (10^−9^ s) and high reactivity.

The central source of H_2_O_2_ is enzymatically catalyzed by superoxide dismutation through the enzyme superoxide dismutase SOD. SOD is the only enzyme that can clear **^•^**O_2_^−^ and it is present at the mitochondrial level as well, in the cytoplasm and extracellular space [99]. It is composed of three isoforms, SOD1 (Cu/Zn-SOD is the predominant **^•^**O_2_^−^ scavenger and is localized in the cytoplasm), SOD2 (Mn-SOD, in the mitochondrial intermembrane space, nucleus, and lysosomes) and SOD3 (Cu/Zn-SOD, is localized in the mitochondrion and extracellular matrix) [100].

**Figure 4:**
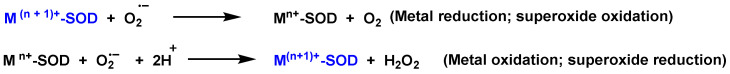
SOD-catalyzed dismutation of the superoxide radical. M = [Cu (*n* = 1); Mn and Fe (*n* = 2)]. The oxidation state of the metal cation varies between n and n + 1.

SOD-catalyzed dismutation of the superoxide radical can be characterized as the next half-reactions, Figure 4.

Oxidative burst is the rapid release of ROS from different cell types, macrophages and neutrophils are especially implicated, and it requires a 10-to-20-fold increase in oxygen consumption through NOx activity. The oxidative burst in phagocytes is commonly associated with bacterial killing, but in the case of alveolar macrophages, they typically produce lower levels of ROS than neutrophils and may require their activation to exhibit their bactericidal properties. Instead, their transient oxidative burst regulates the inflammatory response by inducing cytokine synthesis for redox signaling, resulting in an influx of activated neutrophils and macrophages [101].

In adaptive immunity, ROS-mediated T-cell activation has been suggested to have an immunosuppressive role. T cell activation also requires the help of accessory cells, induction of regulatory T cells Treg by macrophage-derived ROS suppresses other T cells also via ROS. Additionally, localized ROS production drives Treg lineage commitment, while their removal decreases the balance of Treg/T effector cells [102].

## 4. Role of Nitric Oxide Radical ^•^NO and Peroxynitrite ONOO^—^ on Innate Immunity

Nitric oxide synthases NOS are a family of enzymes that catalyze the production of nitric oxide radical **^•^**NO from the amino acid L-arginine [103].

**^•^**NO synthesis has been identified in mammals, fish, birds, invertebrates, and bacteria and there are several NOS isoenzymes [104]. Endothelial eNOS and neuronal nNOS are controlled by calmodulin and, under stimulation, the inducible isoform of iNOS is involved in the immune response, producing large amounts of -NO as an immune defense mechanism [105]. The expression of iNOS is inducible by cytokines (IFN-γ, TNF-α, and IL-2) [106]. All NOS isoforms contain the important cofactor tetrahydrobiopterin THB, and in its absence, NOS produces superoxide ^•^O_2_^−^ instead of **^•^**NO [107]. The iNOS and nNOS isoforms are soluble and predominantly they are found in the cytosol, while eNOS is membrane-associated [108].

iNOS produces large amounts of **^•^**NO as a defense mechanism and is synthesized by several cell types in response to cytokine release and is an important factor in the body’s response to parasite attack, bacterial infection, and tumor growth [109]. It is the cause of septic shock and plays a role in many diseases of autoimmune etiology [110]. The main **^•^**NO-emitting cells are macrophages, neutrophils, monocytes, and mature dendritic cells, by expressing the iNOS [111]. When iNOS is activated, it induces a large, sustained **^•^**NO production, greater than that of the constitutive forms [112].

In adaptative immune regulation, **^•^**NO inhibits the lymphocytes T helper 1 (Th1 is a population characterized by the release of IL-2 and IFN-γ) and promotes Th2 lymphocytes, which leads to humoral immunity and allergic responses [113]. Increased iNOS expression in T cells also regulates Th17 cell differentiation [114].

Induction of iNOS usually occurs in the presence of an oxidative environment, which produces peroxynitrite when **^•^**NO reacts with superoxide [115], leading to cellular toxicity. These features may define the roles of iNOS in host immunity, allowing its involvement in antimicrobial and antitumor activities by defense cells. Also, **^•^**NO inhibits the expression of the inflammatory cytokines IL-1β, TNF-α, IL-6, IFN-γ [116] in lymphocytes, eosinophils, and monocytes, in an effect mediated by nitrosylation of the transcription factors JAK/STAT (Janus Kinase/Signal Transducer and Activator of Transcription) and NF-κβ [117].

When macrophages encounter foreign molecules, they are activated by up-regulation of NF-kβ, which, together with JAK-STAT-induced inflammatory cytokines, increases iNOS expression and **^•^**NO production [118]. **^•^**NO can lyse various bacteria, viruses, fungi, and parasites due to the formation of products such as peroxynitrite, NONOates, S-nitrosothiols, and nitrous acid [119]. In the skin, **^•^**NO provides a protective barrier against micro-organisms, and regulates melanogenesis and the formation of erythema that can occur from exposure to ultraviolet UV light [120].

**Figure 5:**
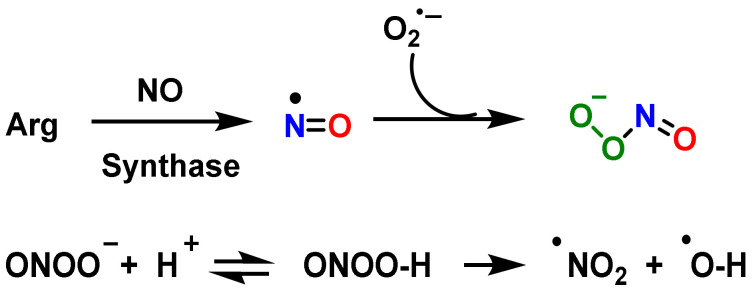
Up: Reaction of radical ^•^NO and anion ^•^O_2_^−^, producing peroxynitrite. Down: Decomposition of peroxynitrite to ^•^NO_2_ and ^•^OH.

During an infective process, macrophages and other innate immunity cells especially produce ROS and RNS (^•^O_2_^−^, ^•^NO and ONOO^—^), but excessive production can cause injury and toxicity to host cells [121]. Fortunately, these host cells are equipped with a protective mechanism called the antioxidant defense system, which protects macrophages and other uninfected cells from the toxic effects of RNS [122]. Peroxynitrite is synthesized from the reaction of ^•^NO with ^•^O_2_^−^, Figure 5 up. The protonated form of ONOO^—^ (ONOOH, pKa = 6.5 to 6.8) decomposes rapidly to RNS, yielding approximately 28% of free ^•^NO_2_ and ^•^OH radical [123], Figure 5 down.

Following induced inflammation, neutrophils and then monocytes infiltrate the affected tissue. Neutrophils kill bacteria and infected cells and induce tissue destruction and cell apoptosis, while monocytes differentiate into macrophages at the same site. Macrophages and other phagocytes eventually eliminate apoptotic neutrophils, contributing to the resolution of inflammation. Leukocyte infiltration is mainly controlled by chemokines and their production is positively or negatively regulated by iNOS-derived **^•^**NO. Part of the mechanisms underlying these dual effects of **^•^**NO remain unknown, so the level of **^•^**NO expression and the duration of **^•^**NO exposure appear to be determining factors. The production of pro-inflammatory cytokines appears to be actively suppressed by TGF-β and NO, produced by phagocytes interacting with apoptotic cells. In summary, **^•^**NO plays an important role during inflammation and is a potential target for therapeutic development in inflammatory diseases [124].

In response to inflammatory signals, macrophages increase the production of cytokines and **^•^**NO, which is important for pathogen clearance. During pro-inflammatory circumstances, also referred to as “M1”, macrophages undergo several metabolic changes, including the reconnection of their tricarboxylic acid (TCA) cycle. **^•^**NO, through its interaction with heme and non-heme metal-bound proteins, together with components of the electron transport chain, functions as a regulator of cellular respiration and as a modulator of intracellular cellular metabolism. The role of **^•^**NO in macrophage reprogramming has been known for a long time, but current models greatly underestimate its importance [125].

The phagosome environment is acidic and reactive nitrogen and oxygen species are generated there, providing a redox chemical environment, which becomes the primary fight against infection. Fluctuations in RNS and ROS levels induce other phases of the immune response. **^•^**NO activates specific signal transduction pathways in tumor cells, endothelial cells, and monocytes, regardless of their concentration. As ^•^O_2_^−^ can react directly with ^•^NO, forming peroxynitrite, the bioavailability of ^•^NO and thus its responsiveness is modified. The ^•^NO/ROS balance is also important during the transition from Th1 to Th2 [125,126].

Microbial clearance depends on **^•^**NO concentration [127], which in turn depends on NOS activity and the availability of arginine in phagosomes. Nicotinamide adenine dinucleotide phosphate NADPH and superoxide act synergistically in phagosomes to scavenge **^•^**NO and increase peroxynitrite levels which, in an acidic environment, contribute to the “cauldron effect” [128]. This mechanism is important during phagocytosis, as the acidic environment of phagosomes provides the right conditions to produce RNS leading to the lysis of pathogens [129]. The “cauldron effect” into the phagosome occurs as it provides an “isolated” environment for the cell to carry out the “destruction” of foreign bodies. ROS, **^•^**NO, and RNS interact to trigger redox reactions. The concentration of NO in a phagosome may depend on the type of NOS in the vicinity and its activity, as well as other localized cellular factors. **^•^**NO and its metabolites, such as nitrites and nitrates, together with ROS, combine forces to kill pathogens in the acidic environment of the phagosome.

**^•^**NO and RNS eradicate bacteria by nitrosylation and oxidation of bacterial macromolecules [126]. Greater bactericidal activity is observed when **^•^**NO and H_2_O_2_ act synergistically, compared to **^•^**NO alone [130]. Lysis of *Staphylococcus* bacteria involves the sequential exposure to respiratory burst followed by **^•^**NO exposure [126]. *Mycobacterium tuberculosis* is killed by **^•^**NO produced by macrophages, the acidic environment of alveolar phagosomes favors the generation of RNS from **^•^**NO, causing oxidation of the methionine residue of enzymes present in the bacteria [131]. It also protects T cells from *Mycobacterium*-mediated apoptosis and supports disease eradication from adaptive immunity.

Not all bacteria are killed by **^•^**NO, but their infectivity is always reduced. Elevated levels of **^•^**NO cause bacterial lysis through nitrosation of the thiol groups of proteins responsible for the formation of pores in the cell membrane, thus preventing the bacteria from leaving the infected cell and preventing the spread of infection to other cells [132].

NO is also an important element in the control of viruses such as rhinovirus, cytomegalovirus, herpesvirus, vaccinia virus, etc. [133]. Most of these viruses cause the induction of iNOS via the Toll-like receptor-3 [134]. **^•^**NO causes nitrosation of the cysteine residues of essential proteins for viral replication [135]. Examples of these proteins are integrases and nucleocapsid proteins, and after being nitrosated they are unable to bind to DNA and cannot function as topoisomerases, thus preventing the integration of viral DNA into host cell DNA [128]. In contrast, during viral infection, high levels of **^•^**NO can produce some undesirable effects in the patient, such as hemorrhagic fever [136].

Human parasites subjected to removal using **^•^**NO/ONOO^—^ include Plasmodium, Leishmania, and Toxoplasma, the most common one being the Plasmodium infection (malaria). Increased production of **^•^**NO in malaria infection, according to Kun and Weinberg et al., 2001, is associated with a single nucleotide polymorphism of the iNOS gene promoter sequence called NOS2 lambarene (G954C) mutation [137]. Leishmania is also successfully destroyed by **^•^**NO [138], as well as inhibiting Toxoplasma infectivity and preventing disease progression to other systems, especially the central nervous system CNS [139]. **^•^**NO is also involved in the immunity provided against Giardia intestinalis, in the study by Zarebavani et al., 2017, a strong increase in **^•^**NO and its derivatives was observed among patients infected with this parasite [140].

In recent years, several studies have linked nitric oxide as a key mediator in numerous neurodegenerative diseases, such as Parkinson’s disease (PD), Alzheimer’s disease (AD), amyotrophic lateral sclerosis (ALS), Huntington’s disease (HD) and ischemic brain injury (stroke). **^•^**NO, alongside its numerous physiological functions (as a mediator of blood vessel dilation, neurotransmitter, and neuromodulator), can be converted into highly reactive and toxic molecules that readily react with proteins, DNA, and lipids to alter their function. This dual action makes it a contributing player in pathophysiology, but also makes the development of effective treatments for neurodegenerative diseases particularly difficult [141,142,143,144,145].

## 5. Role of Hypochlorous Acid HOCl on Innate Immunity

**Figure 6:**
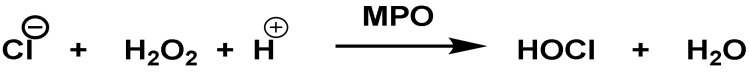
Catalyzed conversion of H_2_O_2_ and chloride to HOCl.

The enzyme myeloperoxidase MPO is expressed in neutrophil granulocytes (a subtype of white blood cells) and produces hypohalous acids to carry out its antimicrobial activity [146]. The most common of these acids is hypochlorous acid, an important inorganic bactericidal molecule present in the innate immune system, which is effective against a wide range of micro-organisms [147]. During the oxidative burst process, cells utilize O_2_ and convert it to ^•^O_2_ using the mitochondrial-membrane–bound enzyme NOx [148] and after to H_2_O_2_ through a family of enzymes called superoxide dismutase SOD [149]. Then, the myeloperoxidase enzyme catalyzes the reaction between H_2_O_2_ and Cl^−^ to generate HOCl [150], Figure 6.

HOCl is the major strong oxidant produced by neutrophils and a potent microbicidal agent. It has been estimated that, in vitro, one million stimulated neutrophils can produce 0.1 μM of HOCl and this concentration can kill 15 million Escherichia coli bacteria in less than 5 min [151]. HOCl reacts rapidly with several biological molecules, particularly those with thiols, thioethers, heme proteins, and amino groups, and can cause tissue injury [152]. A non-essential amino acid such as taurine, at a concentration of 15 mM, is found naturally in cells and acts as a scavenger of HOCl, buffering collateral damage to cellular macromolecules (proteins, lipids, and DNA) [153]. Taurine present in human plasma and cells and HOCl form taurine chloramine (-O_3_SCH_2_CH_2_NHCl), a more stable and weaker oxidant [154]. Tau-NHCl, which accumulates in the extracellular medium and does not inhibit neutrophil functions, may continue to moderate neutrophil cytotoxic activity long after HOCI has been eliminated.

HOCl demonstrates broad-spectrum antimicrobial activity from 0.1 to 2.8 μg/mL concentrations [155,156], Table 1. MBC means Minimum Bactericidal Concentration in μg/mL.

Basic physicochemical properties of HOCl are relevant to its endogenous physiological function including its role as an innate immune factor, topical antimicrobial, and environmental toxicant [157]. HOCl is a weak halogen-based acid and a powerful chlorination agent, that contains one labile proton (pKa = 7.46) dictating the co-existence between acid and conjugated base under physiological conditions at near equimolar ratio. HOCl and its conjugated base HOCl/OCl^−^ represent a potent oxidizing redox system [E0′ = +0.9 (OCl-); E0′ = +1.48 V (HOCl)] under physiological conditions, and serves as an endogenous microbicidal agent, generated by myeloid lineage-derived effector cells (including neutrophils) [157]. The oxidant couple HOCl/OCl^−^ is an endogenous microbicidal agent [158]. Myeloperoxidase produces HOCl and yet other hypohalous acids such as hypobromous acid HOBr, hypoiodous acid HOI, and hypothiocyanous acid HOSCN as an essential component of antimicrobial innate immunity [159]. Endogenous hypohalous acids, while functioning as agents in innate host defense, can also induce tissue damage at sites of inflammation, an area of research in the context of neurodegenerative disorders, Alzheimer’s and Parkinson’s diseases, metabolic and cardiovascular dysfunction (atherosclerosis, diabetes), autoimmune dysregulation and ageing, among other conditions. The MPO system is also implicated in various skin pathologies, such as hypersensitivity and contact irritation, psoriasis, UV damage, photoaging, and melanoma [160].

On the treatment of viral infections, Yamamoto et al., 1991, show already at that time that the influenza virus IFV was inactivated by MPO-myeloperoxidase treatment of human polymorphonuclear leukocytes in the presence of H_2_O_2_ and that viral protein modification occurred in all major proteins, including the inner envelope proteins [161]. Tomas Strandin et al., in 2018, found that neutrophil activation products: myeloperoxidase, and neutrophil elastase, along with interleukin-8 (the major neutrophil chemotactic factor in humans), were strongly elevated in the blood of patients with hantavirus HFRS, factors that correlate positively with renal dysfunction [162]. Shubham Shrivastava et al., 2021, maintain that elevated levels of neutrophil-activated proteins such as DEFA1 alpha-defensins, calprotectin S100A8/A9, and MPO myeloperoxidase are associated with disease severity in patients with COVID-19 [163]. A similar idea is maintained by Pravin T Goud et al., in developing an analysis implicating reactive oxygen species and myeloperoxidase in clinical deterioration and mortality in COVID-19 [164].

The disinfection of drinking water supplies by HOCl chlorination can be considered one of the most important milestones in public health [165]. At the same time, HOCl is the microbicidal principle released by standard disinfectants in swimming pools and is widely used throughout the world. Pool disinfection is an essential barrier to prevent the spread of germs, ensuring a healthy and non-infectious pool environment. In recent years, the use of sodium dichloroisocyanurate as an organic precursor to HOCl has emerged, but HOCl/OCl^−^ remains the preferred active microbicidal agent [166].

## 6. Role of Hypothiocyanite OSCN^−^ on Innate Immunity

Hypothiocyanite OSCN^−^ is an antimicrobial agent, the first product of the peroxidase catalyzed oxidation of thiocyanate SCN^−^ by H_2_O_2_. SCN^−^ is considered a pseudohalide (pseudohalogen compounds are polyatomic analogues of halogens, with similar chemistry, replacing them in biological reactions), produced endogenously as a product of the reaction between cyanide (CN^−^) and thiosulfate (S_2_O_3_ ^2−^), in the liver. Sources of CN^−^ include vitamin B_12_ metabolism and foods containing cyanogenic glycosides, e.g., nuts (almonds) and cruciferous vegetables (such as *Brassica*, a genus of plants of the family *Brassicaceae*, such as cauliflower and broccoli). SCN^−^ is abundant in physiological fluids, especially in mucous membranes (saliva, tear fluids, breast milk, and in the mucous layer of the lung), with a concentration of SCN 10 to 100 times higher than in blood. In tobacco-derived smoke, HCN can also be found in concentrations above 200 mg/cigarette, so some smokers may have saliva concentrations of SCN^−^ as high as 6 mM while non-smokers have concentrations of 0.5–2 mM, therefore a high concentration of SCN^−^ in the saliva is a biomarker of tobacco exposure or smoke in a work environment. Thiocyanate is present at levels of 20-120 µM in serum and extracellular fluid and 800–806 mM in saliva (the wide range is attributed to smoking) [167,168].

The MPO enzyme uses H_2_O_2_ to oxidise chloride, bromide Br^−^, iodide I^−^, and thiocyanate SCN^−^, taking them to their respective hypohalogenated acids [169]. At physiological pH, plasma ion concentrations are ~100 mM Cl^−^, 20–100 μM Br^−^, <1 μM I^−^, 20–100 μM SCN^−^, and MPO primarily generates HOCl and HOSCN. HOI formation is usually insignificant due to the low plasma levels of I^−^, although it is generated rapidly, varying significantly with diet (lower levels are detected in vegans than in vegetarians). HOBr is usually formed at low concentrations, it is pH dependent, and also can be improved by food supplement [169].

The relative specificity constants for chloride, bromide, and thiocyanate are 1:60:730, respectively, with thiocyanate being the most favoured substrate for MPO. In the presence of 100 mM Cl^−^, MPO catalyses the hypothiocyanite production at thiocyanate concentrations of 25 µM. At 100 µM thiocyanate, roughly 50% of peroxide is transformed into hypothiocyanite, and the quantity of hypohalogenic acid production was equal to the sum of the individual rates obtained when each of these negative ions was present alone. The percentage of MPO-induced H_2_O_2_ loss in the presence of 100 mM Cl^−^ doubled when 100 µM thiocyanate was added and was maximal at 1 mM thiocyanate. This indicates that at plasma concentrations of thiocyanate and chloride, myeloperoxidase is far from saturated. Thus, thiocyanate is an important physiological substrate of MPO [170,171].

**Figure 7:**
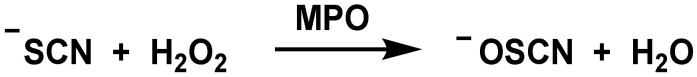
Catalyzed conversion of H_2_O_2_ and SCN^−^ to OSCN^−^.

In general, SCN^−^ is not considered a biologically functional ion, but it plays an important role as a substrate for peroxidase enzymes, which form an essential part of innate defense, including lactoperoxidase LPO, salivary peroxidase SPO, myeloperoxidase MPO and eosinophilic peroxidase EPO [172]. These peroxidases catalyze the oxidation of halides (Cl^−^, Br^−^, I^−^) and pseudohalides (SCN^−^) by H_2_O_2_, resulting in potent oxidizing agents that exhibit antimicrobial activity in vitro and in vivo, as they damage vital structural and functional components of microorganisms. They oxidized various acceptor molecules, e.g., thiocyanate SCN^−^ to hypothiocyanite OSCN^−^, bromide Br^−^ to hypobromite BrO^−^ and iodide I^−^ to hypoiodite IO^−^, Figure 7.

Conversion of SCN^−^ to HOSCN by MPO is reported to be an important detoxification mechanism, by removing oxidant H_2_O_2_ and subsequent HOCl. However, HOSCN also triggers the erythrocytes lysis and perturbs cellular signalling in macrophages and endothelial cells. This process can lead to apoptosis and the discharge of inflammatory mediators, playing a role in the disease pathogenesis.

MPO/H_2_O_2_/OSCN^−^ pathway inhibits bacterial growth, mainly in saliva and breast milk and the lysis mechanism is through inhibition of bacterial glycolysis, targeting glycolytic enzymes with thiol groups (such as glyceraldehyde-3-phosphate dehydrogenase GAPDH, hexokinase HK, glucose-6-phosphate dehydrogenase GPDH, and aldolase), and compromises the capacity of bacteria to transport glucose and other essential nutrients, associated with structural impairment of GLUT transporters or cell membrane. HOSCN/OSCN^−^ is also a potent antiviral and antifungal agent. In contrast to HOCl, HOSCN appears to be innocuous to cells, particularly to cells associated with the oral cavity and airway.

The efficacy range of OSCN^−^ on microorganisms is really wide, including Gram-positive and -negative bacteria (e.g., *Acinetobacter*, *Aeromonas hydrophila*, *Bacillus*, *Burkholderia cepacian*, *Campylobacter jejuni*, *Capnocytophaga ochracea*, *Corynebacterium xerosis*, *Enterobacter cloacae*, *Escherichia coli*, *Haemophilus influenzae*, *Helicobacter pylori*, *Klebsiella*, *Legionella* spp., *Listeria monocytogenes*, *Micrococcus luteus*, *Mycobacterium*, *Neisseria* spp., *Pseudomonas*, *Salmonella* spp., *Selenomonas sputigena*, *Shigella sonnei*, *Staphylococcus*, *Streptococcus agalactiae*, *Streptococcus*, *Wolinella recta*, *Xanthomonas campestris*, *Yersinia enterocolitica*), viruses (e.g., Echovirus, Herpes simplex virus HSV, Influenza virus, Human immunodeficiency virus HIV, respiratory syncytial virus RSV) and yeast and moulds (e.g., *Aspergillus niger*, *Botryodiplodia theobromae*, *Byssochlamys fulva*, *Candida albicans*, *Colletotrichum*, *Fusarium*, *Rhodotula rubra*, *Sclerotinia* spp.).

In the context of the SARS-CoV-2 coronavirus, the etiological agent of COVID-19, the role of hypothiocyanite has been investigated. Several studies maintain that the lack of the LPO/H_2_O_2_/SCN^−^ pathway in nasal and ocular human secretions may explain the survival, proliferation, and environmental dissemination of some bacteria and viruses [173]. A laboratory experiment against the 2009 A/H1N1 pandemic influenza virus showed clear virucidal activity of OSCN-, dose-dependent and without any cytotoxic effect. OSCN^−^ is able to oxidize the capsid proteins of respiratory viruses by creating disulphide bonds in the free thiol radicals of proteins [174].

## 7. Antimicrobial Peptides and Induction of ROS

Antimicrobial peptides AMPs are an important component of the innate immune system in all organisms, including plants, animals, and humans, providing a rapid and non-specific response to pathogens [175]. Cathelicidins and defensins are a family of antimicrobial peptides that show a wide spectrum of antimicrobial activity against bacteria, enveloped viruses, and fungi [176]. Many antimicrobial peptides are stored in the lysosomes of macrophages and polymorphonuclear leukocytes, where they are part of the oxygen-independent activity against pathogens [177,178].

Cathelicidins, first discovered in bovine neutrophils by Zanetti et al. in the early 1990s, are cationic and amphiphilic peptides consisting of 12–97 amino acids, found in humans, other vertebrates and some species of fish [179]. The term cathelicidin was coined from the name cathelin, due to a cathelin-like domain present in cathelicidins. Cathelin itself was coined from cathepsin L inhibitor in 1989. The mechanism of action that triggers the action of cathelicidin involves the disintegration of cell membranes through the formation of pores. This mechanism is common to that of other antimicrobial peptides. Antibacterial, antifungal, and antiviral effects have been observed in cathelicidins. Cathelicidins rapidly destroy lipoprotein membranes of microbes enveloped in phagosomes after fusion with lysosomes in macrophages [179,180].

Most cathelicidins are linear peptides with 23–37 amino acid residues and fold into amphipathic α-helices. Their structure can also be small (12–18 residues) with beta-hairpin structures, stabilized by one or two disulphide bonds. Larger cathelicidin peptides (39–97 amino acid residues) also exist and they have repetitive proline motifs, forming extended polyproline-like structures. The cathelicidins family shares primary sequence homology with the cystatins, of the cysteine proteinase inhibitor family, although amino acid residues considered important in these cystatins are often missing [180,181].

It has been proposed that the primary action of cathelicidins is to induce the production of ROS that damages bacterial molecules, resulting in slowed growth or cell death [182]. Given their low circulating levels in vivo, AMPs may serve to slow the spread of bacterial populations so that the cellular immune system can respond to and fight infection. Unlike atopic dermatitis, skin infections rarely occur in psoriasis due to the presence of antimicrobial agents, including cathelicidin LL-37. LL-37 stimulates the generation of ROS in neutrophils and this process is mediated by a flavoenzyme (most probably NOx) and via an increase in intracellular Ca^2+^ concentrations [183]. Other authors have suggested the role of the human cathelicidin on the activation of ROS/NF-κβ/IL-6 and propose that it could regulate oxidation signaling in cardiovascular diseases like atherosclerosis [184].

Melittin is known in the scientific community as a membrane-active AMP. Recently, a unique antibacterial mechanism for melittin has been proposed. Melittin-induced production of ROS seems to be crucial for the development of apoptosis, in which ^•^OH plays an essential role [185]. Similarly, it has also been suggested that accumulation of ROS and mitochondrial membrane damage may be key for Papiliocin, a novel cecropin-like AMP able to induce fungal apoptosis [186]. Coprisin, a defensin-like 43-mer peptide with three disulfide bonds [187], has been suggested to be a crucial participant in reactive oxygen species (ROS), especially hydroxyl radicals (^•^OH) [188].

Antioxidant functions have also been described for cathelicidins. Recently, a cathelicidin molecule (ARGKKECKDDRCRLLMKRGSFSYV) from the spot-bellied plateau frog *Nanorana ventripunctata* has been shown to reduce the effects of ultraviolet B (UVB) on skin photoaging in mice by ROS scavenging [189]. This could lead to the notion that antimicrobial peptides are involved in the regulation of oxidative-antioxidant homeostasis.

## 8. Mechanisms Developed by Microorganisms to Avoid the Reactive Species

Bacteria contain protective proteins that can detoxify ROS, however, if stress is severe, bacteria can also use ROS to self-destruct. Bacteria regulate the expression of antioxidant defense networks [190]. ROS response is under the control of the master regulators and transcription factors such as OxyR, a positive regulator of H_2_O_2_-inducible genes [191] (i.e., *in S. enterica*, *Francisella tularensis*, and *Porphyromonas gingivalis*), PerR, a ferric uptake regulator (i.e., in *S. aureus* and *Bacillus subtilis*), OhrR, an organic peroxide sensor and transcription repressor (i.e., in *B. subtilis* and *Mycobacterium smegmatis*), and SoxRS, a redox-sensing (i.e., in *E. coli* and *S. aureus*) can be stimulated by direct oxidation of their sensor proteins and then regulating the bacterial comeback appropriately [192]. In general, these regulons control genes needed for antioxidant defense, such as superoxide dismutase, catalase, thioredoxins, heme biosynthesis machinery, glutathione reductases, ferric uptake regulator (Fur), ferritin, and bacterioferritin, i.e., a veritable arsenal whose function is to reduce and cancel out oxidative stress.

Iron homeostasis is critical to mitigate the redox damage induced by the Fenton reaction. Therefore, in pathogenic bacteria *E. coli, S. aureus,* and *Salmonella*, iron-responsive transcriptional repressors can be used to maintain redox homeostasis by controlling the expression of genes encoding iron acquisition systems and iron-dependent enzymes [193].

Metabolism adaptions also play a pivotal role in mitigating oxidative damage, reducing oxidative burden by retarding respiration. For instance, the glyoxylate shunt GS is an anaplerotic reaction of the tricarboxylic acid TCA cycle developed in numerous species, which bypasses two NADH-generating steps [194].

Antioxidant replenishment can also be achieved by metabolic modulation, with the pentose phosphate pathway PPP being an important target for mitigating ROS damage, as NADPH is an antioxidant cofactor. In *E. coli*, by increasing the abundance of glucose-6-phosphate dehydrogenase, metabolic flux can be diverted to the PP pathway, leading to increased tolerance to ROS [195].

Alongside antioxidant defence, activation of oxidative defence regulators is also necessary for the full virulence of pathogens. For instance, OxyR contributes to the virulence of *E. coli* and *P. aeruginosa*. Similarly, SoxRS has been shown to be a positive regulator of Salmonella virulence and pathogenicity (SPI)-2 in *S. enterica* [196].

Certain pathogens exploit ROS to manage their metabolism in order to thrive. For instance, *S. typhimurium* makes use of host-derived ROS during intestinal inflammation. ROS generated by phagocytes converts thiosulphate to tetrathionate, which can be used as a respiratory electron sink by *S. typhimurium*, thus overcoming native microbiota [197].

ROS are key weapons employed by host cells, but they can also induce antibiotic tolerance during infection. ROS generated by macrophages targets the enzymes TCA aconitase and succinate dehydrogenase and force S. aureus into a respiration-reduced metabolism, incompatible with the clearance mechanism of most bactericidal antibiotics [198].

As a summary of the above, ROS are attractive weapons to be used to kill pathogenic microbes. However, they are a double-edged sword and must be regulated with care, as under non-lethal levels of ROS, certain pathogens have evolved mechanisms to adapt and thrive, and may even use ROS as a cornerstone for increasing tolerance and resistance to antibiotics. Targeting bacterial adaptive pathways, along with the use of novel ROS-inducing antibacterial strategies, may be promising approaches to antibacterial therapy [5].

## 9. Conclusions

The innate immune system is the first line of defense against infection and is characterised by a rapid, non-specific response through the production of reactive species (oxygen, nitrogen, and chlorine), which are widely distributed in effector cells (neutrophils, macrophages, etc.) and are biocidal for pathogens. These reactive species are short-lived and are biochemically synthesized by various enzymatic reactions. Cellular antioxidant systems control their physiological levels and play an important role in cell signaling and proliferation. However, at high concentrations and during prolonged exposure, they can combat infectious pathogens by damaging microbial biomolecules such as lipids present in their membrane, making this mechanism a powerful biocidal system. The phagocytic NOx complex generates superoxide anion, a precursor for the synthesis of hydrogen peroxide and in turn hypochlorous acid. Alternatively, the innate system produces reactive nitrogen species through the synthesis of peroxynitrite and its derivatives, which also act as highly microbicidal agents. Deficiency of these antimicrobial agents is associated with severe recurrent infections and immunocompromised diseases, such as chronic granulomatous disease. Ultimately, reactive species play an important and positive role in human health and innate immunity.

## Figures and Tables

**Table 1:** Antimicrobial activity of HOCl.

Bacteria	MBC	Bacteria	MBC
*Escherichia coli*	0.7	*Pseudomonas aeruginosa*	0.35
*Staphylococcus aureus*	0.173	*Staphylococcus epidermidis*	0.338
*Micrococcus luteus*	2.77	*Corynebacterium amycolatum*	0.169
*Haemophilus influenzae*	0.338	*Proteus mirabilis*	0.340
*Staphylococcus hominis*	1.4	*Staphylococcus haemolyticus*	0.338
*Staphylococcus saprophyticus*	0.35	*Candida albicans*	2.7
*Klebsiella pneumoniae*	0.7	*Serratia marcescens*	0.169
*Streptococcus pyogenes*	0.169	*Enterobacter aerogenes*	0.676
*Methicillin-resistant* *Staphylococcus aureus*	0.682	*Vancomycin-resistant* *Enterococcus faecium*	2.73
